# Probabilistic Fingermark Quality Assessment with Quality Region Localisation

**DOI:** 10.3390/s23084006

**Published:** 2023-04-15

**Authors:** Tim Oblak, Rudolf Haraksim, Laurent Beslay, Peter Peer

**Affiliations:** 1 Faculty of Computer and Information Science, University of Ljubljana, 1000 Ljubljana, Slovenia; 2 Joint Research Centre, European Commission, 21027 Ispra, Italy

**Keywords:** fingermark, latent fingerprint, quality assessment, deep learning, quality map, probabilistic interpretation, explainability, forensic, biometrics

## Abstract

The assessment of fingermark (latent fingerprint) quality is an intrinsic part of a forensic investigation. The fingermark quality indicates the value and utility of the trace evidence recovered from the crime scene in the course of a forensic investigation; it determines how the evidence will be processed, and it correlates with the probability of finding a corresponding fingerprint in the reference dataset. The deposition of fingermarks on random surfaces occurs spontaneously in an uncontrolled fashion, which introduces imperfections to the resulting impression of the friction ridge pattern. In this work, we propose a new probabilistic framework for Automated Fingermark Quality Assessment (AFQA). We used modern deep learning techniques, which have the ability to extract patterns even from noisy data, and combined them with a methodology from the field of eXplainable AI (XAI) to make our models more transparent. Our solution first predicts a quality probability distribution, from which we then calculate the final quality value and, if needed, the uncertainty of the model. Additionally, we complemented the predicted quality value with a corresponding quality map. We used GradCAM to determine which regions of the fingermark had the largest effect on the overall quality prediction. We show that the resulting quality maps are highly correlated with the density of minutiae points in the input image. Our deep learning approach achieved high regression performance, while significantly improving the interpretability and transparency of the predictions.

## 1. Introduction

Fingermarks (latent fingerprints) are a special type of friction ridge skin impression, found in unconstrained environments in the scope of a forensic investigation [1]. The deposition of a friction ridge pattern is not controlled, and imperfections are often introduced, which leads to highly inconsistent impressions. Given this complexity, not all impressions can be assigned the same evidential value. Based on the available resources, dactyloscopic experts in forensic laboratories prioritise and filter out impressions of insufficient quality, which tend to be discarded early.

This assessment of quality occurs at different stages during a forensic investigation: (i) The initial decision is made already by crime scene investigators in the field, who determine which marks will be developed and recovered for further processing. (ii) Dactyloscopic experts in the lab would then run automated searches on fingermarks of sufficient quality using an Automated Fingerprint Identification System (AFIS), in an attempt to find corresponding fingerprints in a reference dataset. When choosing a query, the experts would prioritise higher-quality marks based on their previous experience with the particular AFIS. (iii) Finally, the experts determine whether a fingermark–fingerprint pair (resulting from an AFIS search) is suitable for individualisation given the features attributed to both impressions. In practice, the final conclusion would be made by a human expert, who would, upon request, defend it as an expert witness in court.

The assessment of fingermark quality is directly connected to the probability of the successful identification of an individual (given that his/her fingerprints are present in the reference dataset). However, subjectivity and bias can play a role in the decision-making of even highly trained dactyloscopic experts [2,3,4]. This may lead to early rejection of evidence, even if it contains sufficient information for identification. Alternatively, valuable resources could be wasted on impressions with low value for identification. With this work, we aimed to assist the dactyloscopic experts in their decision-making processes using the proposed methods.

In our past research, we developed multiple automated fingermark quality assessment (AFQA) methods [5,6]. These include a classic approach, where specific image- and fingermark-level features were extracted and joined into a 192-value feature vector, as well as a Deep Learning (DL) approach, where the importance of features was determined automatically by a Convolutional Neural Network (CNN). Despite the DL approach performing better and fingermark quality assessment being executed 15-times faster in comparison to the classic approach, one major limiting factor with the DL solution is the transparency of model decisions. While we were able to determine which input features were most important for the classic approach, in our initial work, we did not manage to correlate the predictions of deep models with any particular feature of the input image. Just like an expert witness needs to explain his/her decision before a judge, the assisting automated tools that are used in the course of a forensic investigation should be transparent and backward-traceable in a way that would offer the reasoning behind the predictions. Probabilistic reporting of forensic evidence has existed for a while [7,8], and the European Network of Forensic Science Institutes (ENFSI) is actively promoting probabilistic-based evidence reporting with a set of best practice manuals since 2016 [9]. Due to this, our new deep learning AFQA method with improved result reporting coupled with transparent decision-making should present a great benefit to the scientific community.

**Figure 1:**
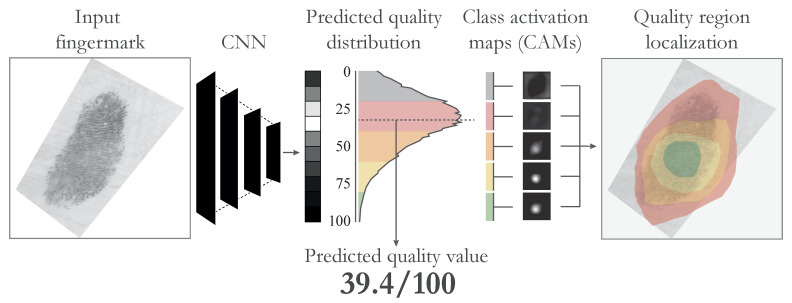
The proposed approach. We used a Convolutional Neural Network (CNN) trained on quality annotations provided by 10 dactyloscopic experts to predict a quality probability distribution as a first intermediate step. From this distribution, we derived the final quality value, as well as the uncertainty of the model and, consequently, the complexity of the input image. Furthermore, we used Class Activation Maps (CAMs) to localise image regions, which contributed most to the specific quality ranges in the predicted quality distribution. The figure is best viewed in colour.

In this article, we present our research on explainable fingermark quality assessment methods. A visual demonstration of the approach is shown in Figure 1. Overall, we made the following contributions:

- We used data produced in the context of a JRC fingermark quality annotation campaign [10] in which 10 international dactyloscopic experts provided quality labels of selected fingermark images from the NIST SD301 and SD 302 datasets [11,12]. We describe the creation of ground truth fingermark quality labels for training the deep learning models.
- We present a novel approach to fingermark quality assessment. We reformulated the problem from a regression task to a probability distribution learning task. The final quality value was produced by calculating the expected value of the predicted quality probability distribution.
- We used GradCAM [13] from the family of eXplainable AI (XAI) techniques to produce Class Activation Maps (CAMs) and interpret them to visualise the connection between the predicted fingermark quality and the input image. Based on our results, the generated quality maps were good indicators of minutiae point density in the input image.

In Section 2, we summarise the state of automated fingermark quality assessment and mention some inspirations from the field of XAI. In Section 3, we present the main contribution of this work, the next iteration of the AFQA models, probabilistic AFQA (pAFQA). Finally, we describe the experimental setup and present the results in Section 4 and provide concluding remarks in Section 5.

## 2. Related Work

We divided the related work on AFQA methods into two groups based on the underlying methodology, the heuristic and data-driven approaches. Then, we discuss various concepts from the field of XAI and introduce them into the domain of fingermark quality assessment.

### 2.1. Automated Fingermark Quality Assessment Methods

With the intention of standardising fingerprint quality assessment, the National Institute of Standards and Technology (NIST) developed the NIST Fingerprint Image Quality (NFIQ) algorithm [14,15,16]. NFIQ was the first quality assessment algorithm to indicate the probability of finding a matching print using an Automated Fingerprint Identification System (AFIS). Automated methods specifically aimed at the evaluation of fingermark quality only started to appear in the last decade in response to rapid digitisation of forensic practices and the need to make the subjective evaluation of fingermark evidence by forensic practitioners more transparent and coherent. A brief overview of these methods is presented in Table 1.

Heuristic approach: Fingermark quality assessment methods in this category use various algorithms for friction ridge processing to extract features and then combine them. Yoon et al. [17] were the first to develop a quality metric specifically for fingermarks, called the Latent Fingerprint Image Quality (LFIQ). They extracted local image features and minutiae data and then heuristically combined them to evaluate the quality of fingermarks. The method was designed under the assumption that the provided minutiae points are reliable. Consequently, the LFIQ provides the most-accurate results when the minutiae are marked manually by trained dactyloscopic experts. On the other hand, the LFIQ performs sub-optimally when using automated minutiae extractors, since these tend to produce spurious minutiae on noisy fingermarks. Sankaran et al. [18] predicted the quality of a fingermark based on a combination of clarity and quality, derived from local image-level features. Clarity is calculated by using second-order image derivatives, while quality is based on the consistency of the local orientation field. Their approach, however, does not consider second-level friction ridge features, such as minutiae points, often used in fingerprint recognition systems. Swofford et al. [19] recently proposed an approach to assess the reliability of individual minutiae and combine those estimates into a global quality value. Like the LFIQ, our tests indicated that their method is sensitive to spurious minutiae and is, therefore, not suitable to be used in combination with automated minutiae extractors.

Data-driven approach: Data-driven methods typically make use of supervised ML techniques and require a set of labelled data in order to guide the optimisation process. The FBI published a series of publications on the topic of evaluating expert opinions [3,4,24,25], where they studied the consistency, variability, and bias of their decisions. In collaboration with an external contractor, the FBI developed the LQmetric [20]. The LQmetric first calculates the minutiae points using an automated minutiae extractor. Then, a local clarity map is constructed using a random forest model, trained on clarity maps that were annotated by dactyloscopic experts. It is these features that are used to predict an overall quality value. The process was further fine-tuned with the result from the FBI’s Next Generation Identification AFIS. The LQmetric is included in the Universal Latent Workstation software, available upon request to law enforcement agencies [23]. Chugh et al. [21] trained a model for quality assessment of fingermarks by using crowd-sourced data, gathered from selected fingermark examiners using a web-based annotation tool. They correlated the annotated labels with a collection of extracted features to determine which features were the most-indicative of fingermark quality. Features with the highest correlation were used to train a quality-assessment model, which yielded a better prediction performance in comparison to the LFIQ. Deep learning was first used for the purpose of fingermark quality assessment by Ezeobiejesi et al. [22]. In their approach, they first segmented the friction ridge impression from the background and then classified individual local patches into different quality classes. The final quality values were determined based on voting on individual patches. In our previous research [6], we compared the performance of “classic” machine learning models to the performance of modern deep learning models in the context of fingermark quality. The superiority of deep models was hindered only by the lack of transparency in the resulting predictions. This paper aims to address this issue.

Many methods in this category rely on the informal “good”, “bad”, and “ugly” labels to classify fingermarks into different quality levels. This labelling scheme was introduced in the (now discontinued) NIST SD 27 dataset [26]. We instead conformed to the standard set by the ISO/IEC 29794-1 [27], which defines biometric sample quality as a value in the range from 1 to 100.

### 2.2. Explaining Model Predictions

Machine learning models, in particular deep neural networks, tend to be rather complex with a substantial parameter space. Due to this, it is very difficult to interpret their predictions, and the models are often considered as black-box solutions. In recent years, more focus is being directed towards explainable deep models.

Probabilistic reporting: One aspect of making model predictions more transparent is to expand the scope of the results, available to the final user. We were inspired by the recent developments in the field of Blind Image Quality Assessment (BIQA). BIQA methods aim to evaluate image quality in general and are used for many practical applications, such as remote imaging, compression, enhancement, etc. An approach currently popular with BIQA methods is predicting a quality probability distribution, which can be interpreted using different statistical measures to derive the final quality value. Liu et al. [28] first used a CNN to extract a latent feature vector from an input image. The authors then used a separate model, called the Label Distribution Support Vector Regressor (LDSVR). The LDSVR is a multi-output support vector machine, which predicts a target quality probability distribution. Similarly, Zeng et al. [29] proposed a probabilistic model for BIQA; however, they trained a CNN to predict a probability distribution vector in an end-to-end fashion. In the training stage, the loss function was calculated using the Kullback–Leibler (KL) divergence [30], which minimises the difference between the predicted and target quality probability distributions. In general terms, these approaches can be considered as label distribution learning [31], since multiple labels are estimated at the same time. This concept is also particularly useful in our case, since the ground truth data for a specific fingermark in our dataset is not a single quality number, but an ensemble of subjective scores from different fingermark examiners. In contrast to simply calculate the average, or Mean Opinion Score (MOS) [32], a distribution of quality values also encodes other properties, such as variance or skewness. These offer better insight into the disagreement of the expert crowd and, consequently, the complexity of the fingermark in question.

Calculating attribution: One category of XAI approaches tries to solve the outcome explanation problem. These methods provide an interpretable connection, called attribution, between an input instance (e.g., a fingermark image) and the model prediction (e.g., a quality score) by following how information propagates through the network during computation [33,34]. Class discriminative localisation maps have become a popular method for explaining deep models in recent years. Zhou et al. [35] were the first to propose an approach to generate Class Activation Maps (CAMs) for networks with a global average pooling layer. They weighted the activation maps of the last convolutional layer by activations from the last fully connected layer to calculate the CAM for a specific input. Selvaraju et al. later generalised this approach and proposed GradCAM [13], an algorithm that uses the backpropagation of gradients to weigh the activation maps. This means that any network can be used to calculate CAMs and a global average pooling layer is not required. GradCAM has been criticised for sometimes showing irrelevant regions as important due to its averaging step. HiResCAM [36] attempts to address this by using elementwise multiplication of the feature maps and gradients instead of only using the average gradient. Muhammad et al. [37] argued that CAM methods often imply that classification is 100% correct when calculating CAMs. They proposed EigenCAM, which instead computes the principal components of the learned feature maps. These methods offer a way to connect the predicted quality of a fingermark with the specific pixels or pixel regions in the input image. Since determining the quality of fingermarks on a continuous scale from 0 to 100 is not a classification problem, we need to modify the problem definition and change the underlying methods to enable the usage of CAM methods.

## 3. Probabilistic Fingermark Quality

In this section, we first define the problem, then we describe the CNN architecture used in our experiments, and finally, we describe our approach to explain the individual predictions of the model.

### 3.1. Problem Formulation

To calculate a quality value y∈[1,100] from an input image $x\in R^{n}$, in this article, we propose a CNN learning strategy such that the learned model $F_{CNN}$ produces quality values that are as close as possible to the ground truth quality labels *y*:

(1) $$\hat{y}=E\left(\hat{q}\right),\;\;\;\hat{q}=F_{CNN}\left(x;\theta_{CNN}\right),$$

where $\hat{y}$ is the predicted quality value for an input fingermark image *x*. $F_{CNN}:R^{n}\mapsto R^{100}$ is a CNN model with $\theta_{CNN}$ being its “learnable” parameters. The CNN outputs an intermediate prediction $\hat{q}\in R^{100}$, which is a vector representing the discrete quality probability distribution in a range from 1 to 100. To calculate the final predicted quality value for a fingermark, we simply take the expected value (or mean) of the predicted probability distribution:

(2) $$E\left(\hat{q}\right)=\sum_{i=1}^{100}i\times {\hat{q}}_{i},$$

where i∈[1,100] represents individual quality bins and each $q_{i}$ is equal to the probability $P(q_{i}=i)$ that the fingermark falls into that bin, i.e., has a quality of *i*.

The predicted probability distribution allows us to compute various distribution properties. Here, we used the expected value as our final quality value; however, other statistics could be used instead. For example, if the predicted quality distribution is skewed, a distribution median might be more useful as the final quality value.

Finally, we search for the optimal parameters θ of the model with the following loss function:

(3) $$L\left(q,\hat{q}\right)=\frac{1}{m}\sum_{i=1}^{m}q_{i}\times \log\left(\frac{q_{i}}{{\hat{q}}_{i}}\right),$$

which minimises the difference between the predicted quality distribution $\hat{q}$ and the ground truth quality distribution *q* using the Kullback–Leibler divergence [30].

### 3.2. CNN Encoder

As the basis for our predictive model $F_{CNN}$, any modern CNN architecture can be used. We retained the configuration of the initial convolutional layers of the reference architecture. Based on preliminary testing, we used two fully connected layers with 256 neurons after the convolutional layers, both followed by a Leaky ReLU [38] activation function. The selected activation function retains the good convergence performance of the established ReLU function, but also guarantees non-zero gradients during training. Finally, an output layer with 100 neurons and a softmax activation is added to produce a quality probability distribution vector $\hat{q}$ with 100 values that sum up to 1. The hierarchical structure and the widening of perceptual field allows the model to learn image features for fingermarks captured at different scales, which means predictions can be made on fingermark images of varying resolution (PPI).

### 3.3. Generating Target Probability Distributions

A major novelty of this approach lies in the prediction of an intermediate probability distribution $\hat{q}$ before a final quality value is determined. In order to train the model in a supervised manner, we first need to produce the target probability distributions *q*. For a particular fingermark image, we are given quality labels $y_{l}$ from a set of *L* grading functions. While we use quality labels annotated by an ensemble of 10 dactyloscopic experts [10], the approach is general and allows for any kind of ensemble of ground truth scores to be used. Instead of simply computing the Mean Opinion Score (MOS), we modelled a discrete normal distribution for each of the labels $y_{l}$ and then joined them together into a final probability distribution *q*. The equation for individual probability $q_{i}$ for a given quality value *i* is then

(4) $$q_{i}=\frac{1}{L}\sum_{l=1}^{L}N\left(i;y_{l},\sigma \right),\;\;\;i\in \left[1,100\right].$$

**Figure 2:**
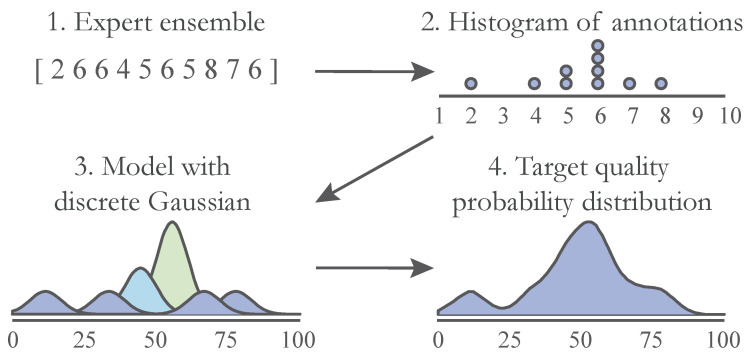
Creating the quality probability distributions. Here, we show the process of constructing the quality probability distribution from multiple labels, gathered from a group of 10 dactyloscopic experts [10]. For each label in a range from 1 to 10, we model the normal distribution and then add them together to generate a final quality distribution of the fingermark.

Here, σ is a parameter for the standard deviation of the normal distribution N. We assumed equal σ for all grading functions and set it to 1. However, if the uncertainty of the individual labels is known (for example, if an examiner is less confident in his/her decisions), we can modify this parameter accordingly. The process of creating the target probability distributions is shown in Figure 2.

### 3.4. Calculating Attribution

We wanted to be able to interpret the predictions of the model and better understand what it actually learned. The first step is to choose the appropriate XAI approach for our problem. The aspect we were most interested in is feature attribution. Specifically, we aimed to establish a connection between the output of the model and the input fingermark image. In other words, we would like to calculate the contribution of individual pixels to the final quality prediction. To calculate feature attribution, we used CAMs. While our problem is not a classification task by definition, the output layer of the CNN encoder allowed us to treat it like one. We can use the following process:

(5) $$c_{i}=G\left(x;i\right),$$

where $G:R^{n}\mapsto R^{n}$ can be any CAM-generating algorithm, *x* is the input image, i∈[1,100] is a specific quality value (or class), for which we want to generate the CAM, and $c_{i}\in R^{n}$ is the resulting CAM. For a single fingermark image and a quality range from 1 to 100, we obtained a set $C\in R^{100\times n}$ of 100 CAMs, which were generated based on the final convolution layer of the network.

### 3.5. Quality Region Localisation

Finally, we propose two ways of interpreting the calculated attributions for individual quality values. The first interpretation is an overall contribution map. Here, we joined all contributions over the entire quality spectrum by adding the individual CAMs in *C* into a single map $c_{sum}=\sum C\in R^{n}$. The resulting heat map indicates which regions in the image have the largest overall impact on the final prediction. The higher the value of the individual pixels, the more important these are during inference.

**Figure 3:**
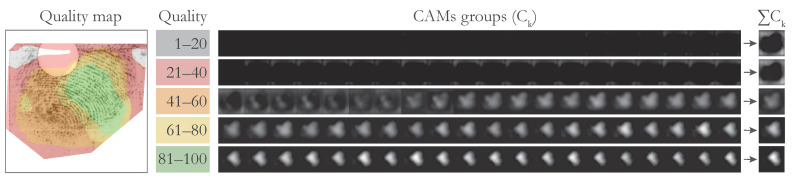
Creating the quality map. Groups of Class Activation Maps (CAMs) are joined together to calculate the contribution of individual pixels towards different quality values in the distribution. The quality spectrum is divided into 5 quality ranges, which correspond to the 5 colours with which we visualise the computed regions: grey, red, orange, red, and green. The figure is best viewed in colour.

The second interpretation is a quality map. We wanted to calculate how much individual pixels contributed to a specific sub-range of the final quality spectrum. To achieve that, we joined together *K* groups of consecutive CAMs in *C*. The parameter *K* determines the number of quality levels, which will be assigned to individual pixels. The choice of *K* is arbitrary and can be changed based on the requirements of the final system. In our approach, we used K=5 to ensure a direct comparison and to maintain compatibility with some previously established quality metrics (friction ridge quality is commonly divided into 5 quality levels in the related literature [14,20]). We split *C* into groups $C_{1}=\left\{c_{1},c_{2},\cdots ,c_{20}\right\}$, $C_{2}=\left\{c_{21},c_{22},\cdots ,c_{40}\right\}$, $C_{3}=\left\{c_{41},c_{42},\cdots ,c_{60}\right\}$, $C_{4}=\left\{c_{61},c_{62},\cdots ,c_{80}\right\}$, and $C_{5}=\left\{c_{81},c_{82},\cdots ,c_{100}\right\}$. For each group $C_{k}$, we added together the contained CAMs into a single map $\sum C_{k}$. The resulting maps $\sum C_{k}$ thus show pixel contribution towards a specific range of quality. The process of obtaining maps $\sum C_{k}$ is shown in Figure 3. Finally, for each pixel in the input image, we found the $C_{k}$ where the contribution of that pixel was the strongest: $c_{quality}=\arg\max_{k}\sum C_{k}$. The resulting map $c_{quality}\in Z^{n}$ specifies a quality level of each respective pixel in the input image. A value of 1 means that the pixel contributed most positively towards the lowest quality range (1–20). In contrast, a value of 5 means the pixel contributed most positively towards the highest quality range (80–100).

## 4. Experiments

In this section, we first describe the experimental setup, datasets, and metrics. Then, we discuss the quantitative results and compare our model to existing quality assessment methods. Finally, we show how our interpretation of feature attribution correlates with actual friction-ridge-level features.

### 4.1. Data

We used two fingermark datasets, namely the NIST SD 302 [12] and NIST SD 301 [11]. Both datasets contain fingermarks lifted from various surfaces by trained forensic examiners in a simulated environment. In total, the datasets contain 11200 fingermarks along with rolled and flat fingerprints from 224 subjects.

In order to integrate expert opinion into our AFQA models, we organised an annotation campaign in June 2022, where 10 experts assessed the quality of a collection of friction ridge impressions [10]. The certified dactyloscopic examiners were invited from 8 member states of the European Union, Australia, and Europol. During 6 one-hour sessions, the experts used a web-based annotation tool to assign quality values in a range from 1 to 10 to a collection of fingermark images. In total, we gathered quality labels for 956 fingermark images with varying quality and 44 images of rolled fingerprints, which represent the best-possible quality impressions in the subset. In this way, the model was exposed to the entire quality spectrum of friction ridge impressions during training. On average, each fingermark received a quality score from 8 distinct examiners during the annotation campaign. We used these quality annotations to train our models in a supervised manner.

For the purpose of this paper, we used two subsets of the NIST SD301 and SD302 datasets. The data were divided as follows:

- Training data: These included the aforementioned set of 1000 annotated images, coming from both the SD301 and SD302. For each image and the respective examiner annotations, we created a discrete quality probability distribution using Equation (4) to be used as the target during training. The training data were used for the initial model selection by performing a 10-fold cross-validation. For each fold, 10% of the data were reserved as the validation set. Once the best model configuration was found, the entire training set was used to train the final model.
- Test data: A hold out set of 9115 images from the SD302 dataset alone was used to compare the final model to the state-of-the-art and to demonstrate our approach to quality region localisation. The images were selected to ensure that there was no overlap with the training set. Out of the 9115 images in the test set, 6665 also included minutiae point annotations, which were recently added to the SD302 dataset by NIST.

Given the relatively small size of the annotated training set, we used data augmentation to artificially enlarge the set during training. We carefully selected specific image manipulation techniques, which did not interfere with the friction ridge pattern in the input image. We used the following operations: flip vertically (p=0.5), flip horizontally (p=0.5), random rotation in a range of [−90, 90] degrees, and random translation by 5% of the image size. By doing this, we wanted to ensure that the quality predictions were invariant to the rotation or small translation of the friction ridge pattern while retaining all the existing information contained in the impression. Furthermore, the images were padded to maintain the aspect ratio of the captured impression. Finally, all images were resized to a resolution of 512×512.

### 4.2. Metrics

To assess the regression performance of our models, i.e., to measure the ability to learn the given task, we used standard regression metrics. These included the Mean-Squared Error (MSE), Mean Absolute Error (MAE), which have to be minimised, and the R-squared ($R^{2}$), which needs to be maximised. These metrics were also used to perform the initial model selection. Furthermore, we used two correlation metrics to compare our model with other existing friction-ridge-quality-assessment models. We used the Pearson Linear Correlation Coefficient (PLCC), which measures the linear correlation between two sets of quality scores. Since the relation between two metrics is not guaranteed to be linear, we also calculated the Spearman Rank Correlation Coefficient (SRCC). The SRCC measures how two variables are monotonically related and is therefore less sensitive to outliers or non-linear relationships.

### 4.3. Experimental Setup

For the selection of the backbone CNN model, we first performed a 10-fold cross-validation on the training set. In the end, we selected ResNet [39] as the architecture of choice, in particular because it offers a good compromise between regression performance, execution time, and model size. We have shown in our previous research [6] that a depth of 34 layers was sufficient for ResNet to capture features related to friction ridge quality. During training, we used the Adam [40] optimisation algorithm with a learning rate of $1\times 10^{-4}$, which was multiplied by a factor of 0.1 on the loss plateau. In order to facilitate a faster optimisation process, we initialised the network with the weights pre-trained on the ImageNet [41] dataset. This resulted in a significantly faster convergence compared to using random weights, while at the same time, this did not have a negative effect on the final performance of the model.

To generate the CAMs, we opted for a gradient-based approach, which follows the propagation of gradients through the network to determine the contribution of input pixels to a specific class in the output vector. We also considered various perturbation-based methods [42,43], which modify the input to calculate the contribution. These methods, however, dramatically increase the computational complexity of the system due to the many forward passes needed to generate a detailed activation map. The implementation of the CAM-generating algorithm was provided by Captum [44], an interpretation framework for PyTorch. Specifically, we used the GradCAM [13] implementation. We also tested other iterations of the algorithm, such as HiResCAM [36] and GradCAM++ [45], but found no significant added value to the final attribution maps. GradCAM generates the attribution for a specific layer in the target network. We chose the final layer of the ResNet-34 to be the target layer from which the attribution was calculated. The final layer of the encoder typically learns the more high-level concepts within the provided data. Due to the down-sampling that occurs in the CNN, the resulting CAMs had a reduced resolution of 16×16. We used bi-cubic interpolation to resize the CAMs to the original image size of 512×512.

To train and test our models, we used an Ubuntu workstation with a GeForce RTX 3080 GPU. With this configuration, using a ResNet-34 as the backbone, the model computes the quality score for a single image in 5 milliseconds on average. Furthermore, it takes around 450 milliseconds to calculate the CAMs and the resulting quality maps.

### 4.4. Quantitative Evaluation

The main reason behind using a discrete probability distribution as the target variable is to obtain a better understanding of the predicted quality value. Besides calculating the final quality value, we can extract other distribution properties, such as the level of uncertainty. In principle, learning to predict a discrete probability distribution is a more complex problem than predicting a single variable. In our first experiment, we explored whether the added computational complexity adversely influences the training process or the final performance of the model more.

In our first experiment, we compared the performance metrics of two models, where one was trained to predict the MOS values and the other was trained to predict the quality probability distributions, from which the expected value from Equation (2) was calculated. Both were trained on the training set using a 10-fold cross-validation. For the probabilistic model, we computed the expected value of both predicted and ground truth distributions and computed the regression metrics between these values. The results are shown in Table 2.

On average, the probabilistic model achieved a KL-divergence of 0.138 between the target and predicted distributions. Once we calculated the expected value, we compared the predictions with the MOS-based model. We observed that predicting a quality distribution had no disadvantages compared to predicting the MOS quality value directly. On the contrary, the probabilistic model appeared to perform better, based on the regression metrics. The $R^{2}$, which indicates the correlation between the ground truth and predicted scores, was relatively high for both models. However, using the probabilistic model, we observed an $R^{2}$ of 0.951 and 0.013, which were higher than the $R^{2}$ of the MOS-based model. We believe the additional information about the spread of scores, embedded within the quality probability distributions, provided better guidance to the training process. This, in turn, resulted in better regression performance. Furthermore, the probabilistic model also achieved lower MSE and MAE metrics. To put the errors in context, in a range from 1 to 100, the resulting MAE represented only around 4% of the whole output range. The MSE, on the other hand, was more indicative of how the models handle outliers, where again, the probabilistic model performed better.

With an $R^{2}$ of 0.951, the probabilistic model achieved a relatively high correlation with the examiner annotations. In our previous research [5,6], we trained several regression models based on quality scores, obtained by existing quality assessment methods, which were trained to predict the results of an AFIS. Although these models were trained on a different subset of the SD301/SD302 dataset, we never observed regression performance as high as that in Table 2. The grading function of the examiner ensemble appeared more linear in relation to fingermark quality and was therefore easier to approximate by a machine learning model. We believe this contrast was caused by two factors related to existing AFQA methods. (a) These methods might only work when all pre-defined conditions are met. For example, an impression will be given a quality score of 0 if the size of its area is below a certain threshold. (b) Some methods use a set of handcrafted features, which might not be sufficient to capture the wide array of distortions present in a fingermark image. Humans, on the other hand, are much better at extrapolating between two concepts and can, therefore, be more consistent even when observing a new one.

**Figure 4:**
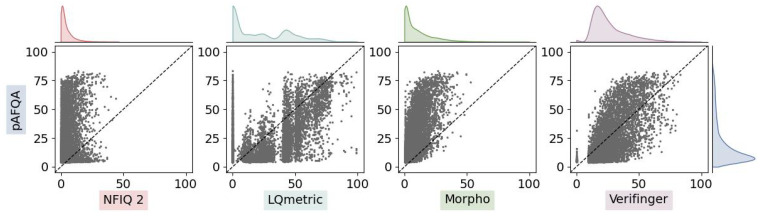
A scatter plot comparing the pAFQA with existing solutions. We compare visually a selection of quality assessment methods, computed on the testing subset of the NIST SD302 dataset. Each dot in the plot represents one fingermark image, where the *y*-position marks the pAFQA quality values, while the *x*-position marks the quality values of other quality metrics. Displayed also are the score distribution histograms of the five quality metrics being compared.

We compared the pAFQA with four other methods, namely (i) the open-source fingerprint quality metric NFIQ 2, (ii) the LQmetric, a quality assessment method used by the FBI, and the metrics (iii) Verifinger and (iv) the Morpho quality metric, provided by two commercial vendors in their fingerprint matching software packages. Note that the NFIQ 2 and both commercial methods were trained on different data for different purposes. Furthermore, NFIQ 2 was developed solely on AFIS performance predictions, while the LQmetric predictions relied on manual annotations from dactyloscopic experts. We assumed that Verifinger and Morpho were developed to predict matching performance for AFIS solutions, but have no information regarding their design. In contrast, the pAFQA model in this paper was trained solely on expert annotations. The comparison can be observed in Figure 4. We visualise the scatter plots between the metrics together with the respective score distributions. The visualisation is complemented with correlation metrics, shown in Table 3.

Within the group of evaluated quality assessment methods, the NFIQ 2 had the lowest correlation with the pAFQA by far. Given that the NFIQ 2 was trained on flat fingerprints, a big difference between pAFQA and NFIQ 2 was expected. This was confirmed by the low correlation, which means that the pAFQA and, by extent, the expert opinion on fingermarks could hardly be approximated with NFIQ 2, or vice versa, even if accounting for potential non-linearity and different scales of the effective output range. The remaining quality metrics were much more correlated with the pAFQA. A similar behaviour to that of the NFIQ 2 quality algorithm was observed for the Morpho quality metric, where relatively low quality values were attributed to fingermarks (the highest score given was 53). However, it appeared Morpho was much more correlated with the pAFQA if adjusted for scale. Morpho was then followed by Verifinger, which had a similarly non-linear, almost sigmoidal scatter pattern on a considerably larger output range. Third was the LQmetric, which had a much more linearly correlated pattern. We can also observe the ripples in the scatter pattern of the LQmetric, which appeared to be the result of some non-linear decision-making in the core of the algorithm. Overall, the visualisation in Figure 4 demonstrates well the difference between these quality metrics and the variance of the attributed scores. For example, one metric can attribute a very high score (>90), while the other could give a very low score (<10) to the same fingermark image.

### 4.5. Interpreting Probability Distributions

**Figure 5:**
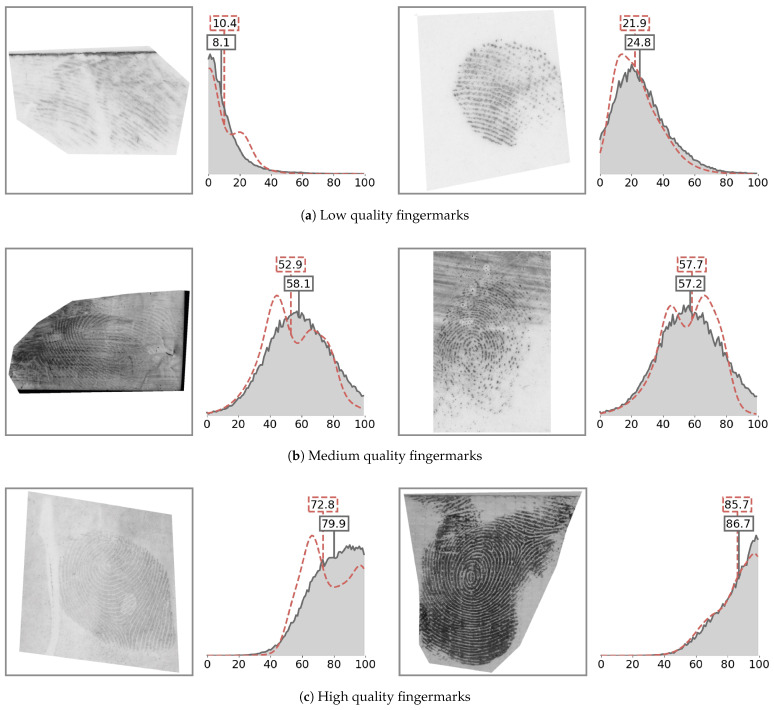
Quality distributions of various fingermarks. Here, we show the predicted quality distributions of (**a**) bad-quality, (**b**) medium-quality, and (**c**) good-quality fingermarks from a validation subset during training. The dashed red line represents the target quality probability distributions, created from expert labels, while the grey line is the predicted quality distribution. Indicated also are the derived final quality values for predicted (grey rectangle) and target distribution (red rectangle).

In this section, we demonstrate how the pAFQA model predicts the intermediate quality probability distributions. These are shown in Figure 5. For each fingermark image, the red line represents the target quality probability distribution, while the predicted distribution is shown in blue colour. We also show the final quality value (expected value of the probability distribution) with the vertical lines, with colours matching their respective distribution.

First, we observed that all fingermarks were assigned a final quality value very close to the target quality value. However, the slight changes between the predictions and ground truth labels only become apparent when comparing the quality distributions. For example, the predicted distributions were mostly Gaussian-like, while the target distributions were often skewed, or sometimes even bi-modal. This came from the variance in the opinions of the expert ensemble, the distribution of which was not necessarily Gaussian. The predictions, however, were mostly normally distributed, which suggested that the model was able to generalise rather than over-fitting to the labels of individual examiners. This made the model more robust and removed the effect of potential outliers. This was particularly visible on medium-quality fingermarks (b), where the examiners often had varying opinions about the quality and the variance of the scores was consequently larger.

A qualitative assessment of the attributed scores revealed several properties of the pAFQA model. First, low-quality fingermarks (a) mostly contained impressions with missing friction-ridge-level features, such as minutiae points. The fingermark on the left appears to have a visible friction ridge pattern; however, the pattern is severely blurred in one direction. This makes the ridges ambiguous, since we cannot be sure whether a ridge structure is real or only a smudge. The fingermark on the right-hand side contains a clearly visible ridge, but the area is relatively small and only contains a few minutiae points. The medium-quality fingermarks (b) caused the most discrepancies amongst the examiners. These often contained a large enough impression, but ambiguous ridges. For example, in the left image, the polarity of ridges changes (on the right, the ridges are lighter than the background; on the left, they are darker). On the right image is a dry impression with many discontinuities in the ridge structure. The high-quality fingermarks (c) often contained visible cores and deltas, as well as a clear ridge structure. Our model was able to attribute high quality values to these images even at different contrasts between ridges and valleys of the impression. In contrast, classic ML models often struggle with low-contrast images.

### 4.6. Attribution-Based Quality Maps

The quality of a friction ridge impression is usually not consistent throughout the entire impression. The impressions tend to have multiple regions with varying quality. For example, half of a fingermark can have perfectly distinguishable ridge formations including second-level detail, while the other half could be severely distorted. Predicting the quality probability distribution provided us the opportunity to see whether the pAFQA was able to differentiate between high- and low-quality regions within a fingermark. To visualise this, we computed the CAMs for each quality value represented by the discrete quality distribution.

#### 4.6.1. Model Focus

**Figure 6:**
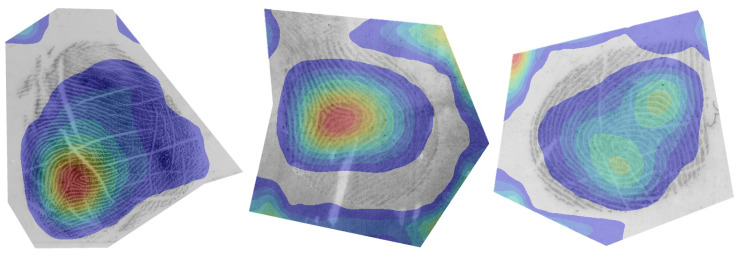
Salient regions on high-quality fingermarks. We can observe how the model focuses on the central area of the fingermark, where core points (loops and deltas) are present. The presence of core points can often be considered as an indicator of a high-quality fingermark. The figure is best viewed in colour. Blue colour indicates a low contribution and red a high contribution toward the final prediction.

**Figure 7:**
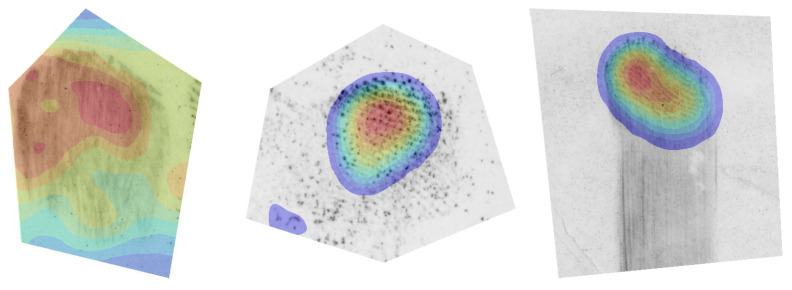
Salient regions on low-quality fingermarks. In the case of low-quality fingermarks, the focus area of the model is spread out, often with no clearly distinguished region of interest. The figure is best viewed in colour. Blue colour indicates a low contribution and red a high contribution toward the final prediction.

In Figure 6 and Figure 7, we observe the overall contribution map, generated using the outputs of the GradCAM algorithm. Such an image would supplement the final quality prediction and would allow an end user to better understand the prediction. Note that the activation maps were much smaller in size compared to the original image due to the hierarchical structure of the CNN. The CAMs were, therefore, resized back to the dimensions of the original input image. Due to this, the resulting maps were not very detailed and, therefore, only had the ability to weakly localise the contribution to the final prediction. The visualisation of CAMs was masked to match with the regions of interest of fingermark images.

We first looked at the visualisations of high-quality fingermarks in Figure 6. The pAFQA model focused on the high-level features of the fingermark, specifically the cores and deltas [46]. These are locations where the orientation of the friction ridge pattern changes. When these features are present in a fingermark, the dactyloscopic experts will consider such traces as high(er)-quality. This is because, from the location of cores and deltas in a fingerprint image, we can determine the first-level detail—the general pattern of the fingerprint, which on its own already contains some evidential value [47]. If a fingermark can be correctly classified into one of the general patterns, this means that we can eliminate fingerprints with a different pattern and the number of possible matches is substantially reduced. Additionally, we also know the orientation, which helps with the matching process, since fingerprints in a reference database are normally oriented upwards from the phalanx in the bottom to the fingertip at the top. The presence of cores and deltas could, therefore, speed up the automated matching process.

In contrast, we can see how the model interpreted a low-quality fingermark image in Figure 7. On fingermarks that contained no clear friction ridge structure, but still contained some high-frequency information, such as dust and other particles, the model focused mostly on the empty space, and the centre of attention appeared to be spread out over the image. On fingermarks that contained clearly distinguishable friction ridge information, the model was able to roughly localise the borders of the impression.

#### 4.6.2. Quality Region Localisation

**Figure 8:**
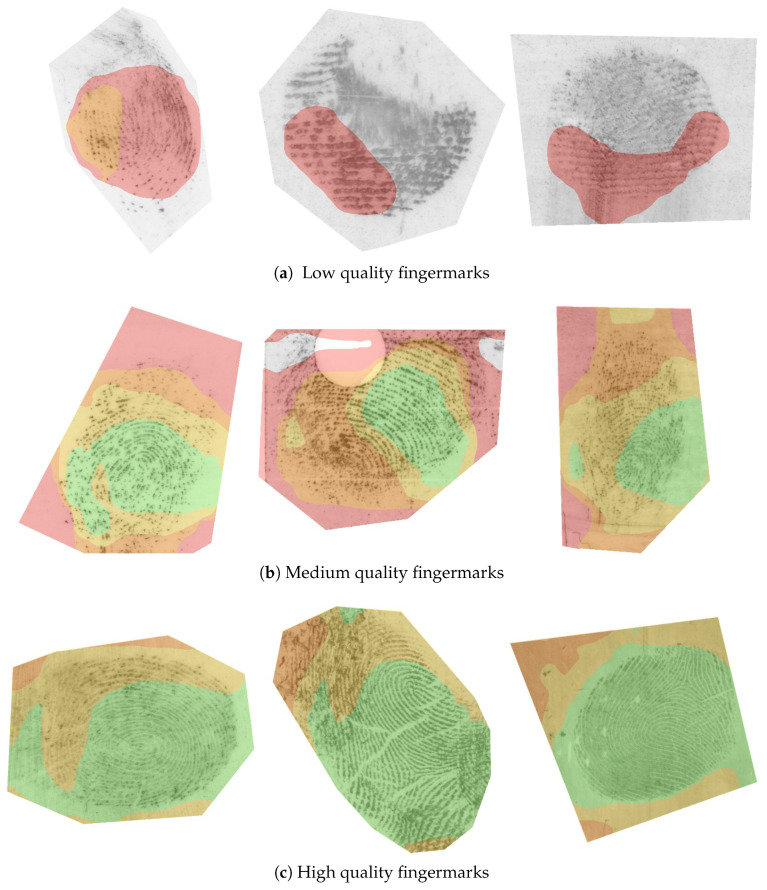
Quality region visualisation. In our final experiment, we show how the CAMs can be correlated with different quality regions in the input fingermark image. The figure is best viewed in colour. The colours represent the respective quality region in a range of 0–20 (grey), 20–40 (red), 40–60 (orange), 60–80 (yellow), and 80–100 (green).

The intensity of individual CAMs is proportional to the contribution of the input pixels towards the respective quality value. We can, therefore, add CAMs from a particular quality range and construct a quality map that visualises the different quality regions of the fingermark. As already indicated in Figure 3, we attributed different colours to the different quality regions: the lowest-quality region (0–20) is coloured grey, low-quality (20–40) red, medium-quality (40–60) orange, high-quality (60–80) yellow, and highest-quality (80–100) green. The final results are presented in Figure 8.

The first row (a) contains only fingermarks with a small impression area, various distortions, or ambiguous friction ridge structure. Consequently, the image pixels are mostly categorised into the lowest- and low-quality regions. We can see that that empty and noisy areas in the image were best correlated with the lowest-quality category. However, we can see that the model appeared to correctly localise the ridge structure in all three cases in the first row. The fingermarks in Row (b) mostly contain a mix of different quality regions. Within the fingermarks in Row (c), we can observe high contributions to the highest-quality range (80–100) for large areas of the impression. This also included the first-level features of the fingermark, i.e., cores and deltas, when these were present. The calculated quality maps may contain some artefacts (apparent salient regions, where there was no friction ridge pattern from that category present). Such artefacts normally occurred on the border of the friction ridge pattern where the image was masked or were a side-effect of resizing the CAMs to the dimensions of the original image. We believe the generated quality map should be observed together with the overall contribution map to obtain the best understanding of the final quality prediction.

#### 4.6.3. Minutiae Point Density

Purely by intuition, the quality maps generated by the pAFQA model appeared to be connected with some aspects of fingermark quality. In this experiment, we established the correlation between the generated quality regions and any friction ridge- or image-level features that are used in practice to individualise friction ridge impressions. Based on the visual analysis of the maps, we already deduced that the generated CAMs showed a strong response around the cores and deltas of the impression. These points are important for the orientation and classification of an impression; however, there is also strong evidence that suggests minutiae points are more densely distributed around those areas in comparison to other areas of the friction ridge structure [48,49,50]. Based on this, we hypothesised that the generated quality maps were related to the minutiae density in the fingermarks.

Another good indicator for minutiae density is local clarity. While clarity by itself does not guarantee the presence of minutiae, it makes it easier for dactyloscopic experts to detect them if they are present [25,51]. The clarity and density of minutiae are therefore highly correlated [18]. We can use this information and examine how well our generated maps predicted the minutiae density, in comparison to a clarity-based map. We used clarity maps produced by the LQmetric [20], which was generated by a random forest model based on clarity annotations from dactyloscopic experts.

**Figure 9:**
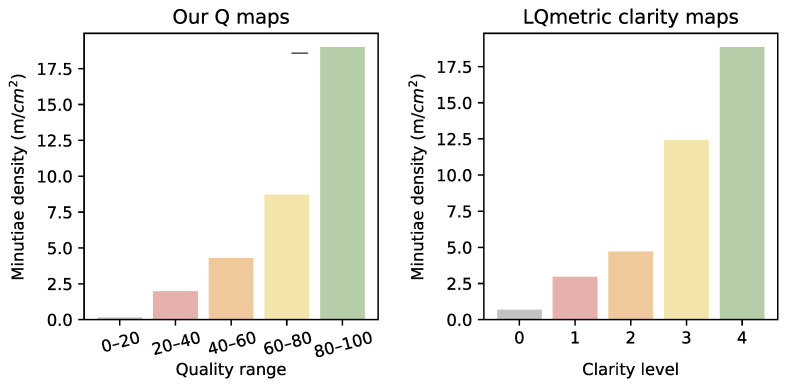
Attribution-based quality maps as indicators for minutiae density. We assessed the the correlation between different quality regions and the density of minutiae (in minutiae/cm^2^) and compared the results with the clarity maps, produced by the LQmetric [20].

To confirm our hypothesis, we measured the minutiae frequency in the fingermarks, present in the SD302 dataset, and associated it with the five quality regions that made up our quality maps (grey, red, orange, yellow, and green). We first calculated the total area of each region produced on our test set. Given the known pixel density (PPI) of the images in the SD302 dataset, we transformed the area measurement from $pixels^{2}$ to cm^2^. Next, we counted minutiae points for each of the five regions independently. Finally, we calculated the minutiae density by dividing the total number of minutiae by the total area for a particular region. We repeated this procedure for the LQmetric clarity maps. The results are shown in Figure 9. We observed that both our quality maps, as well as the LQmetric clarity maps were highly correlated with the minutiae density. In comparison, the quality maps produced by our model appeared to be more consistent; each successive quality region contained roughly twice the number of minutiae of the previous level. The first quality level contained almost no minutiae points and was therefore a good background indicator. For the highest level in both our quality maps and LQmetric clarity maps, the minutiae density was calculated at 19.0 min/mm^2^ and 18.9 min/mm^2^, respectively. The average minutiae density ranges from around 19 to 24 mm^2^ in fingerprints [52]. The results in Figure 9 were consistent with these statistics. Given that even the best fingermark impressions contain some imperfections, we expected the minutiae density in the highest-quality areas in the fingermarks to be slightly lower than that of the fingerprints.

**Figure 10:**
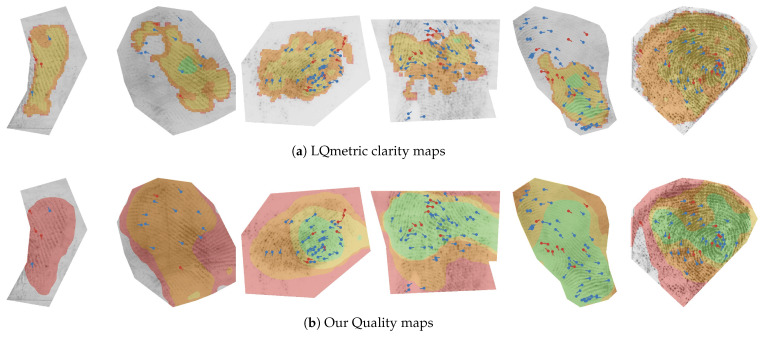
Qualitative evaluation of quality maps. We demonstrate how our proposed quality maps (**b**) visually correlate with the manually annotated minutiae points, provided in the NIST SD302 [12] dataset. Red points represent ridge bifurcations, and blue points represent ridge endings. We compared the results on the same set of fingermarks with the clarity maps, generated by the LQmetric (**a**). The figure is best viewed in colour.

In Figure 10, we show the quality maps in relation to the minutiae points and compare these with the clarity maps produced by the LQmetric. The difference in the density of the minutiae for a specific quality region became apparent once the minutiae were superimposed. The green regions captured well the areas with a high minutiae density, in particular near singular points (cores and deltas). The density then dropped with lower-quality regions. In comparison, the LQmetric clarity maps were more detailed and fit better to the area of the friction ridge pattern, which made it a better tool for determining the region of interest. However, the relation between the LQmetric clarity maps and the minutiae point density was not as apparent visually. We can conclude that the quality maps generated by the pAFQA model were able to roughly localise and identify regions with different levels of minutiae density.

#### 4.6.4. User Presentation

**Figure 11:**
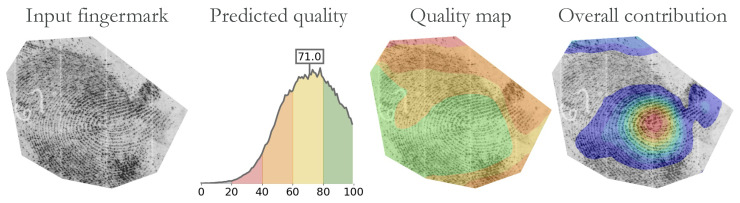
Example of the results, as presented to the end user. The pAFQA method not only predicted fingermark quality, but also generated intermediate representations, such as a quality distribution, a quality map, and the overall contribution of pixels to the final prediction. For the best understanding of model prediction, the results should be viewed together. The figure is best viewed in colour.

The pAFQA method was designed with the intent to assist the dactyloscopic experts in their work. The final quality prediction is best interpreted together with other intermediate results, as shown in Figure 11. We coloured the different sub-ranges of the quality probability distribution to match the colours in the quality map for a more intuitive understanding. The quality map could be used to better guide the dactyloscopic experts when marking friction ridge features. This visualisation can contribute to “more informed” decisions and serve as a transparent bridge between the AI method and the interpretation of forensic evidence.

## 5. Conclusions

In this article, we presented a study on explainable fingermark quality assessment methods using deep learning. We proposed the pAFQA model, which predicts a quality probability distribution as an intermediate result, prior to calculating the final fingermark quality. The quality predictions were further enhanced with additional information, such as the overall contribution map, which showed the contribution of individual pixels to the final prediction, as well as a quality map, which divided the image into different quality regions. This post hoc explanation of model predictions led to a more transparent decision-making and produced results that were interpretable to a human operator. The dactyloscopic experts were, thus, able to connect the predicted quality value with the known visual properties of the friction ridge impression, as they normally would in their standard practice.

Our experiments showed that reformulating the task from a regression problem to a distribution learning problem improved the final regression performance. The properties of the predicted distribution also offered additional information, such as the uncertainty of the model. Finally, we showed that the individual regions in our quality maps correlated highly with the minutiae point density and could be used in practice to better assist forensic experts in their work.

The implementation of the pAFQA was developed based on quality labels, provided by trained dactyloscopic examiners. As the model was trained, tested, and validated exclusively using expert opinion, it is not directly compatible with existing methods, which were developed to produce quality as a predictor of AFIS performance. In order to design a truly universal fingermark quality metric, multiple aspects of fingermark identification need to be considered. Fingermark quality should be indicative of the performance of both human and automated biometric identification systems. In the future, we aim to combine both of these aspects together, as well as develop a common evaluation strategy, which would improve interoperability and enable better comparison with state-of-the-art methods.

## Figures and Tables

**Table 1:** A taxonomy of published related work. These methods are specifically aimed at the assessment of fingermark image quality.

Name	Approach Type	Deep Learning	Target Quality Range	Fully Automated	Implementation Available †
Yoon et al. [17] (LFIQ)	Heuristic	N/A	[1, 100]	No **	No
Sankaran et al. [18]	Heuristic	N/A	Unspecified	Yes	No
Swofford et al. [19] (DFIQI)	Heuristic	N/A	[−1.0, 1.0] for value, complexity, and difficulty	No	Yes
Kalka et al. [20] (LQmetric)	Data-driven	No	[1, 100]	Yes	Yes *
Chugh et al. [21]	Data-driven	No	[1.0, 5.0]	Yes	No
Ezeobiejesi et al. [22]	Data-driven	Yes	Classification into good, bad, and ugly	Yes	No
Ours (pAFQA)	Data-driven	Yes	[1.0, 100.0]	Yes	Yes

† To the best of our knowledge. * Available upon request for law enforcement agencies or for research purposes as part of the ULW [23]. ** Achieves the best performance only using manually labelled minutiae points.

**Table 2:** Comparison between predicting the MOS and probability distribution. We compared the regression performance metrics for two models. The model mMOS was trained as a regressor on the Mean Opinion Score (MOS) of the examiner ensemble, while the mPROB was trained to predict a quality probability distribution. The results indicate that predicting a probability distribution has no adverse effects compared to predicting an MOS.

Model Predictions	MSE	MAE	$R^{2}$	KL-Div
MOS-based	47.49	5.10	0.938	/
probabilistic	**31.25**	**3.98**	**0.951**	0.138

Best regression performance is marked in bold font.

**Table 3:** Correlation between the pAFQA and existing solutions. We calculated Pearson’s Linear Correlation Coefficient (PLCC) and the Spearman Rank Correlation Coefficient (SRCC) to measure the correlation.

Quality Assessment Method	PLCC	SRCC
NFIQ 2	0.209	0.116
LQmetric	0.627	0.591
Morpho	**0.738**	**0.744**
Verifinger	0.730	0.704

Highest correlation to pAFQA is marked in bold font.

## Data Availability

The fingermark images used in this paper have been released publicly by NIST as part of the N2N challenge. Access to the datasets can be requested at https://www.nist.gov/itl/iad/image-group/nist-special-database-302 (accessed on 13 February 2023). The quality annotations that we used to train our models in a supervised manner will be released to the public in the near future. For more information, updates, and the source code for the experiments, visit our GitHub page at https://github.com/timoblak/OpenAFQA (accessed on 13 February 2023).

## Abbreviations

The following abbreviations are used in this manuscript:

AFQA	Automated Fingermark Quality Assessment
MOS	Mean Opinion Score
PPI	Pixels Per Inch
CNN	Convolutional Neural Network
XAI	eXplainable AI
CAM	Class Activation Map
PLCC	Pearson Linear Correlation Coefficient
SRCC	Spearman Rank Correlation Coefficient
MSE	Mean-Squared Error
MAE	Mean Absolute Error
